# Diversity of Root Nodule-Associated Bacteria of Diverse Legumes Along an Elevation Gradient in the Kunlun Mountains, China

**DOI:** 10.3389/fmicb.2021.633141

**Published:** 2021-02-16

**Authors:** Jinfeng Pang, Marike Palmer, Henry J. Sun, Cale O. Seymour, Ling Zhang, Brian P. Hedlund, Fanjiang Zeng

**Affiliations:** ^1^State Key Laboratory of Desert and Oasis Ecology, Xinjiang Institute of Ecology and Geography, Chinese Academy of Sciences, Ürümqi, China; ^2^Xinjiang Key Laboratory of Desert Plant Roots Ecology and Vegetation Restoration, Xinjiang Institute of Ecology and Geography, Chinese Academy of Sciences, Ürümqi, China; ^3^Cele National Station of Observation and Research for Desert Grassland Ecosystem in Xinjiang, Cele, China; ^4^College of Resources and Environment, University of Chinese Academy of Sciences, Beijing, China; ^5^School of Life Sciences, University of Nevada, Las Vegas, Las Vegas, NV, United States; ^6^Desert Research Institute, Las Vegas, NV, United States; ^7^Nevada Institute of Personalized Medicine, Las Vegas, NV, United States

**Keywords:** microbial diversity, endophytes, root nodules, legumes (Fabaceae), elevation, Kunlun Mountains

## Abstract

Bacteria in root nodules of legumes play important roles in promoting plant growth. In this study, we investigated root nodule-associated bacteria isolated from leguminous plants along an elevation gradient on the northern slope of the Kunlun Mountains, China, using a cultivation approach. In total, 300 isolates were obtained from seven legume species within six ecological zones. Isolates were identified based on 16S rRNA gene phylogenetic analysis and potential rhizobia were further identified using a *rec*A gene phylogeny. Among the isolates, *Bacillales* (particularly *Bacillus*) were the dominant isolates from all host legumes and all elevations (63.5%), followed by *Rhizobiales* (13%) and *Pseudomonadales* (11.7%). Less than 3% of the isolates belonged to *Burkholderiales*, *Paenibacillales*, *Enterobacteriales*, *Actinomycetales*, *Sphingomonadales*, *Xanthomonadales*, *Chitinophagales*, *Brevibacillales*, *Staphylococcales*, or *Mycobacteriales*. A few elevation-specific patterns emerged within the *Bacillales* and *Pseudomonadales*. For example, isolates related to the psychrotroph *Bacillus psychrosaccharolyticus* were only isolated from the highest elevation sites (>3,500 m) whereas those related to the mesophile *Bacillus endophyticus* were only isolated from lowest elevation sites (1,350 m), suggestive of a role of soil temperature in their distribution. Similarly, isolates related to *Pseudomonas brassicacearum* were the dominant *Pseudomonadales* isolates, but they were only isolated from middle and low elevations (<3,200 m). A total of 39 isolates belonged to the *Rhizobiales*, 36 of which were confirmed to the genus level using the *recA* gene. In all, *Rhizobiales* isolates were obtained from five different host legumes spanning the entire elevation gradient. Those from the low-elevation Qira Desert-Oasis Transition Zone (1,350–1,960 m) suggested some patterns of host preference. For example, most isolates from *Albizia julibrissin* formed a monophyletic group related to *Rhizobium lemnae* and most from *Alhagi sparsifolia* were closely related to *Ensifer kummerowiae*. In general, this study shows that most bacteria associated with root nodules of legumes are widely distributed in distinct ecological zones within a single geographic region but suggests that both climate and host interactions may influence their distributions.

## Introduction

The Kunlun Mountains fall within a region affected by the Mongolian-Siberian dry anticyclone, also known as the Siberian Mongolia High. This semi-permanent high-pressure system develops early in the winter each year, and spans at its maximum across most of Asia, toward parts of Europe (Yihui, 1990; Guo et al., 1997; Chen et al., 2020). Typically, the stronger the high-pressure system in this area, the colder the winters, while warmer winters in Asia are associated with a weaker high-pressure system (Yihui, 1990; Chen et al., 2020). The northern slopes of the Kunlun Mountains border the Taklamakan Desert, with typical temperatures ranging between −25 and 34°C. The annual precipitation for this region is less than 100 mm and is mainly concentrated in July and August (Bruelheide et al., 2003; Yang et al., 2006). Within this region, the Qira River flows from south to north with a gentle slope. This region has very little precipitation (Xue and Gui, 2015), with very limited vegetation.

The harsh environment on the northern slope of the Kunlun Mountains means that limited plant species can survive under these conditions. However, legumes generally have a high tolerance to stress, including damage caused by wind-blown sand, and can stabilize soils and enhance soil fertility (Esperschütz et al., 2011; Gentzbittel et al., 2015), thus becoming one of the main plant families in the region. Microbial endophytes colonize the internal tissues of living plants without causing discernable harm (De Bary, 1866; Wilson, 1995; Hallman et al., 1997), and can enhance plant growth or survival. Many stress tolerance characteristics of legumes and other plants are conferred or enhanced by either rhizosphere, rhizoplane, or endophytic plant growth-promoting bacteria (Aroca and Ruiz-Lozano, 2009). In these instances, microorganisms can act as probiotics for their plant hosts contributing to host fitness (Ruiza et al., 2011; Hardoim et al., 2015). The plant microbiome can improve plant growth and health, with one of the better understood effects being production of diverse volatile organic compounds that serve as signaling molecules to regulate plant growth and immunity in response to biotic and abiotic stresses (Khalaf and Raizada, 2018).

Among these plant growth-promoting endophytes are rhizobia, which is a collective term for a polyphyletic group of Gram-negative bacteria that can establish symbiotic relationships with leguminous plants (Masson-Boivin and Sachs, 2018; Poole et al., 2018). This association leads to the creation of symbiotic nodules, which provide central carbon metabolic pathway intermediates and a low dissolved oxygen concentration for biological nitrogen fixation to occur (Kuzma et al., 1993). Rhizobia differentiate within the mature nodule and the resulting bacteroids are protected within the root nodules. Rhizobia and host plants exchange a variety of nutrients, including organic carbon, which is fixed by the plant and transported to the microbe, and nitrogen, which is fixed by the bacteroids and transported to plant tissues in the form of ammonia and amino acids. In addition to rhizobia, other symbiotic bacteria, collectively called nodule-associated bacteria, have been isolated from the root nodules of a variety of legumes (Muresu et al., 2008; Dudeja et al., 2012; De Meyer et al., 2015; Zgadzaj et al., 2015).

**FIGURE 1:**
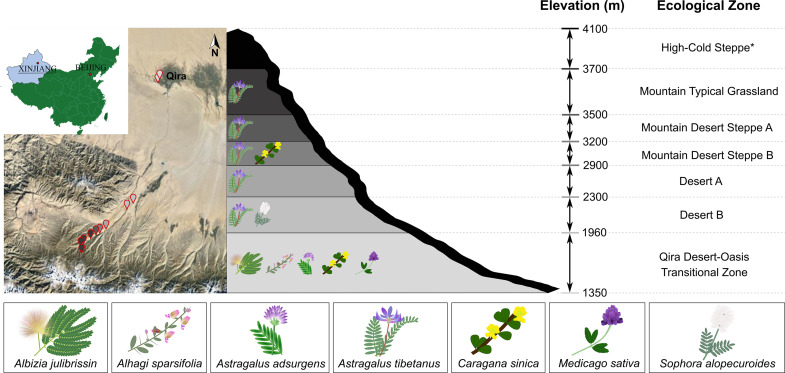
Sampling locations and legumes sampled in the Kunlun Mountains. **Left**, map showing the geographic location of sampling sites on the northern slope of the Kunlun Mountains situated in Xinjiang, China. Sampling points are colored based on the elevation or ecological zone indicated in the schematic representation of the region **(right)**. Legumes were sampled from all ecological zones except for the High-Cold Steppe (indicated with asterisk). Legumes sampled in each ecological zone are indicated in the schematic.

In recent years, there has been increasing interest to characterize and bioprospect endophytes within root nodules in diverse legumes in different regions (Muresu et al. 2008; Kumar et al., 2013; Xu et al., 2014; De Meyer et al., 2015; Beukes et al., 2019). These studies are usually aimed at obtaining potential endophytic inocula and to elucidate functional relationships between endophytes and host plants. Despite this growing interest, very limited studies of bacteria associated with root nodules of legumes in northwest China have been done. Given the extreme conditions of this region, particularly aridity and temperature variability, and a steep elevation gradient instrumented with climate monitoring stations in the Kunlun Mountains, we explored the diversity of bacteria associated with root nodules within six of the seven defined ecological zones in this system, the Qira Desert-Oasis Transition Zone (1,350–1,960 m), the Desert B (1,960–2,300 m) and Desert A zones (2,300–2,900 m), the Mountain Desert Steppe A (2,900–3,200 m) and B (3,200–3,500 m), and the Mountain Typical Grassland (3,500–3,700 m) (Figure 1). All isolates were identified with the 16S rRNA gene and putative rhizobia were further identified and characterized using the *recA* gene and phenotypic analysis. This study forms part of a larger project aimed at identifying high-efficiency nitrogen-fixing strains for agricultural purposes in northwest China.

## Materials and Methods

### Sample Collection, Soil Chemistry, and Climatic Data

Healthy-looking legumes representing members of the *Papilionoideae* (*Astragalus adsurgens*, *Astragalus tibetanus*, *Alhagi sparsifolia*, *Caragana sinica*, *Medicago sativa*, and *Sophora alopecuroides*) and the *Caesalpinioideae* (*Albizia julibrissin*) were selected within six ecological zones (Cui et al., 1988) and 13 sampling locations along the elevation gradient (1,706–3,576 m) within the Qira River Basin (Figure 1). These legumes were removed from the soil using sterile tools, starting from 30 to 50 cm around the main root (or 30–100 cm for phreatophytes in the Qira Desert-Oasis Transition Zone), including the main root and large lateral roots. Multiple root nodules were harvested from each plant (37 total plants) with sterile forceps and placed in separate sterile centrifuge tubes containing autoclaved silica gel, which eliminates condensation and therefore limits microbial growth during storage and transportation (Yates et al., 2004; Ampomah and Huss-Danell, 2011). Centrifuge tubes were stored in the dark in an ice box for transport to the laboratory within 2 days where they were stored in the refrigerator until processing within 48 h. GPS locations of sampling sites are shown in Supplementary Table 1, along with information on the individual microbial isolates.

Rhizosphere soils were collected during excavation of each individual plant (*n* = 37) by gently shaking loosely adhered soil from the roots. Soil samples were transported to the laboratory in Ziploc bags. The soil was weighed before and after air drying, and the water content was calculated. Sieved soil (2 mm mesh) was used for soil organic carbon measurement using the potassium dichromate volumetric method with external heating; total phosphorus was measured following digestion with concentrated sulfuric acid and perchloric acid digestion in an AA3 flow analyzer (SEAL Analytical GmbH, Norderstedt, Germany); total nitrogen was measured following digestion with concentrated sulfuric acid and perchloric acid using the Kjeldahl method; and pH was measured using a PHS-3C pH meter (Shanghai Puchun Measure Instrument Co., Ltd., Shanghai, China). Rhizosphere soil chemistry data associated with each plant are shown in Supplementary Table 2 and summarized in Supplementary Figure 1.

**FIGURE 2:**
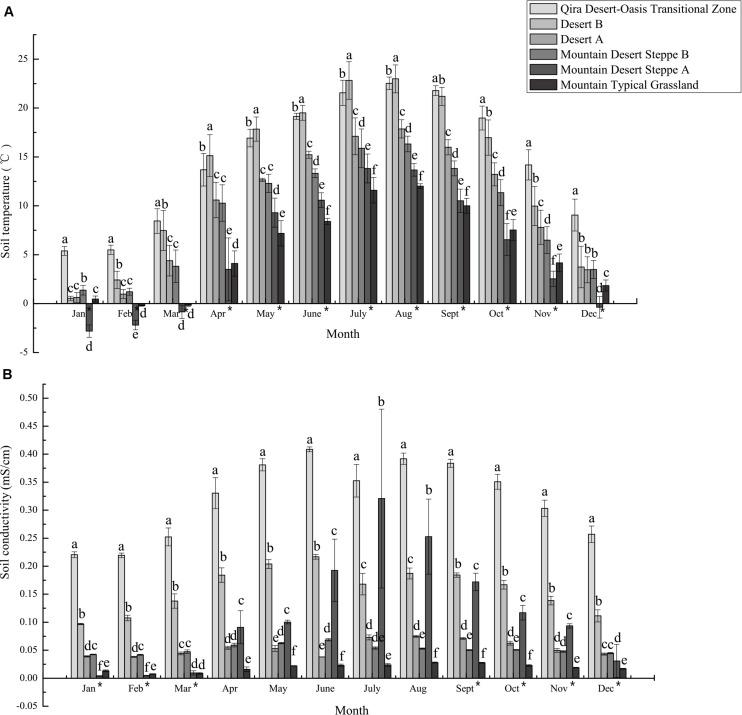
Mean monthly soil temperature **(A)** and conductivity **(B)** measured at climate monitoring stations in the different ecological zones. The bars represent the mean ± S.D. for all data collected for a given month. Asterisks next to months indicate a significant relationship (Spearman’s rho *p* < 0.05) between elevation and soil temperature or conductivity. Distinct letters are significantly different (ANOVA *p* < 0.05).

Climatic and edaphic data gathered over 2018–2019 were provided by Xinjiang Cele Desert Grassland Ecosystem National Field Scientific Observation and Research Station, including atmospheric temperature, relative humidity, soil volumetric water content, soil electrical conductivity, soil temperature^1^ for locations within each ecological zone. Supplementary Table 3 shows monthly mean and standard error from each station. These data, along with statistical relationships between these data and elevation, are plotted in Figure 2 and Supplementary Figures 2–4. Soil measurements are reported here for 1 m depth for the Qira Desert-Oasis Transition Zone and 50 cm depth for all other ecological zones since these are the most relevant for the legumes in those ecological zones and best represents the location of the sampled root nodules.

The mean and standard error of data from both rhizosphere soils and climate monitoring stations were calculated in Excel. These data were analyzed within SPSS Statistics 23 to assess whether values from different ecological zones were significantly different at the time of sampling (for rhizosphere soils) and monthly during a calendar year (for data collected at climate monitoring stations) by ANOVA (two-tailed test) at a probability value of 0.05. Data were visualized with Origin Pro 8 and edited in Inkscape. Additionally, to assess elevational trends in the data, Spearman’s rank correlation coefficients were calculated between elevation and monthly means for both edaphic and climatic data collected during a calendar year using SPSS Statistics 23.

### Isolation, Purification, and Preservation of Bacteria Associated With Root Nodules

All collected root nodules from each plant were hydrated by soaking them in sterile water for 2 h (Yates et al., 2004; Ampomah and Huss-Danell, 2011) and surface sterilization was performed by soaking them in 95% ethanol for 20 s, followed by submersion in 3% sodium hypochlorite for 2–5 min, and rinsing eight times with sterile water. The length of time for sterilization was typically 3 min but it was varied based on the size of the root nodule. For *Albizia julibrissin*, the root nodules had a thick and coarse cortex, so the treatment was lengthened to 5 min. For some small nodules, the length of time was shortened to 2 min to limit exposure of the nodule to hypochlorite. The surface-sterilized nodules from each plant were then crushed with sterile forceps, mixed to generate a composite sample for each sampled plant, and spread evenly onto yeast mannitol agar (YMA) (Pattison and Skinner, 1974) plates and incubated at 28°C. Fast-growing colonies were observed within 3–5 days, and slow-growing colonies were observed after 7–15 days. An effort was made to select a few colonies representing distinct colony morphologies, which is a common approach to maximize diversity sampling (Navarro et al., 2009). Single colonies were then purified by serial streaking onto YMA plates at least three times until a single colony morphology was observed on each plate. Purified strains were stored as frozen suspensions in 30% glycerol at −80°C. While many of these bacteria are likely to be endophytes, the more conservative term “root nodule-associated bacteria” is used here to account for the possibility that rhizoplane bacteria might also have been isolated, particularly endospore formers in the *Bacillales*.

### Genotypic Characterization and Identification

DNA was extracted from bacterial cells grown on YMA plates for 3 days at 28°C by using the TIANamp Bacteria DNA Kit (Tiangen Biotech, Beijing, China) following the manufacturer’s instructions (DP121221). The extracted DNA was dissolved in 50 μL TE buffer and used as the template for PCR. PCR amplification of the 16S rRNA gene was performed by using primers P1 and P6 (P1 forward primer: 5′-CGGGATCCACACTTTGATCCTGGCTCAGAACGAACGCT-3′; and P6 reverse primer: 5′-CGGGATCCTACGGCTAC CTTCTTACGACTTCACCCC-3′) (Deng and Hiruki, 1991), procured from Sangon Biotech (Shanghai, China). Reactions were performed in a BIORAD C1000 Thermal Cycler (Bio-Rad, Hercules, CA, United States) in a total volume of 25 μl consisting of 2 μl of genomic DNA, 10 pmol of each primer, and 2 × Tap PCR Master Mix. PCR was performed under the following conditions: initial denaturation step at 95°C for 5 min, followed by 36 cycles of denaturation at 94°C for 1 min, annealing at 55°C for 1 min, and extension at 72°C for 2 min, with a final extension step at 72°C for 7 min. PCR amplification of the *rec*A gene was performed by using primers *recA*f-63 and *recA*r-504-Meso (*recA*f-63 forward primer: 5′-ATCGAGCGGTCGTTCGGCAAGGG-3′; and *recA*r-504-Meso reverse primer: 5′-TTGCGCAGCGCCTGGCTCAT-3′) (Zhao et al., 2010), procured from Sangon Biotech (Shanghai, China). Reactions were performed in a BIORAD C1000 Thermal Cycler (Bio-Rad, Hercules, CA, United States) in a total volume of 25 μl consisting of 2 μl of genomic DNA, 10 pmol of each primer and 2 × Tap PCR Master Mix. PCR was performed under the following conditions: initial denaturation step at 95°C for 5 min, followed by 36 cycles of denaturation at 94°C for 45 s, annealing at 58°C for 45 s, and extension at 72°C for 1.5 min, with a final extension step at 72°C for 6 min. The amplified PCR products were analyzed by agarose gel electrophoresis and sequenced using the Sanger method using both PCR primers. All information regarding sequences is listed in Supplementary Table 1. Sequences of the 16S rRNA genes were assembled and edited, and initially compared with the National Center for Biotechnology Information (NCBI; United States) refseq_rna database via BLASTn by using the EzBioCloud server^2^ (Chun et al., 2007).

### Phylogenetic Analysis

To compare the sequences obtained from the isolates to known species, BLASTn (Altschul et al., 1997) was used to search the Reference RNA sequences (refseq_rna) and the Nucleotide collection (nr/nt) databases at the National Center for Biotechnology Information (NCBI, United States) for the 16S rRNA and *recA* genes, respectively. The ten most closely related sequences from type strains were retained as references for phylogenetic analyses. These reference sequences were combined with all sequences from the isolates, and duplicate reference sequences were removed from the dataset in BioEdit 7.2.5 (Hall, 1999). For the 16S rRNA gene dataset, multiple sequence alignment was performed through the SINA v. 1.2.11 (Pruesse et al., 2012) web service. In contrast, the *recA* gene dataset was aligned *de novo* using MAFFT v. 7 (Katoh et al., 2019) with default settings. Both the 16S rRNA gene and the *recA* gene datasets were subjected to maximum-likelihood (ML) analysis using RAxML v. 8.2.1 (Stamatakis, 2014) with the General Time Reversible (GTR) model (Tavaré, 1986) and gamma correction for among site variation, while branch support was inferred from 1,000 bootstrap replicates. The phylogenies were visualized with iTOL v. 5 (Letunic and Bork, 2019).

### Phenotypic Characterization

All phenotypic tests were conducted in triplicate at 28°C on YMA plates, unless otherwise noted. Potential nitrogen fixation was tested based on growth on Ashby’s Mannitol Agar. Temperature, pH, and NaCl ranges for growth were assessed on YMA. Carbon source utilization was tested by replacing mannitol in YMA plates with 0.1% of tested carbon sources. Nitrogen source utilization was tested by replacing yeast extract in YMA with 0.1% of tested nitrogen sources.

### Nucleotide Accession Numbers

Near full-length 16S rRNA gene sequences have been deposited in GenBank under accession numbers MT634394-MT634690 and MW396711 (Supplementary Table 1). The sequences for the *recA* gene fragments have been deposited in GenBank under accession numbers MW306700-MW306735 (Supplementary Table 1).

## Results

### Rhizosphere Soil Chemistry, Climate Data, and Legume Distribution in Ecological Zones

The chemical composition of rhizosphere soils in the different ecological zones varied. Organic carbon, phosophorous, nitrogen, and potassium contents were each significantly higher in the high-elevation Mountain Typical Grassland rhizosphere soils compared to other ecological zones (ANOVA *p* < 0.05) (Supplementary Figure 1 and Supplementary Table 2). The pH was circumneutral at all sites and was lowest in the Mountain Typical Grassland.

Similarly, climate and edaphic data collected at research stations over 2018–2019 indicated significant differences between the ecological zones (Figure 2 and Supplementary Table 3 and Supplementary Figures 2–4). Both atmospheric and soil temperatures were negatively correlated with elevation for almost all months (Spearman’s rho, *p* < 0.05), with ≥10°C differences in monthly mean atmospheric temperatures for 6 months (May to October) between the low-elevation Qira Desert-Oasis Transition Zone and the high-elevation Mountain Desert Steppe A, with no significant differences in winter due to atmospheric inversions (Supplementary Figure 2). Soil temperatures at depths relevant to the sampled root nodules showed similar trends, but elevational trends were evident year-round but with lower seasonal variation (Spearman’s rho, *p* < 0.05) (Figure 2A). In January and February, mean soil temperatures were <2.5°C at all elevations except the low-elevation Qira Desert-Oasis Transition Zone, which was significantly warmer than all other soils (ANOVA, *p* < 0.05). Soil conductivity was also negatively correlated with elevation for most months (Spearman’s rho, *p* < 0.05) and was signficantly higher in the low-elevation Qira Desert-Oasis Transition Zone compared to the other ecological zones for all months (ANOVA, *p* < 0.05) (Figure 2B). Soil volumetric water content was highest in the Qira Desert-Oasis Transition Zone and Mountain Desert Steppe A soils for most months (ANOVA, *p* < 0.05), but negative correlations with elevation were only significant for February and March (Spearman’s rho, *p* < 0.05) (Supplementary Figure 3). Soil relative humidity was strongly correlated with elevation for all months (Spearman’s rho, *p* < 0.05), with low-elevation ecological zones having higher humidity in the winter months and high-elevation zones having higher humidity for the rest of the year.

Legumes were most species rich in the low-elevation Qira Desert-Oasis Transition Zone, which hosted five legume species (Figure 1 and Supplementary Table 2). With an increase in elevation, the richness of leguminous plants declined. Five ecological zones hosted *Astragalus tibetanus*: Desert A/B, Mountain Steppe A/B, and Mountain Typical Grassland. *Sophora alopecuroides* only occurred in the Desert B ecological zone, while *Caragana sinica* was only sampled from the Mountain Desert Steppe B and the Qira Desert-Oasis Transition Zone. The High-Cold Steppe was not sampled due to the inaccessability of these high elevations.

### Phylogeny and Distribution of Root Nodule-Associated Isolates

**FIGURE 3:**
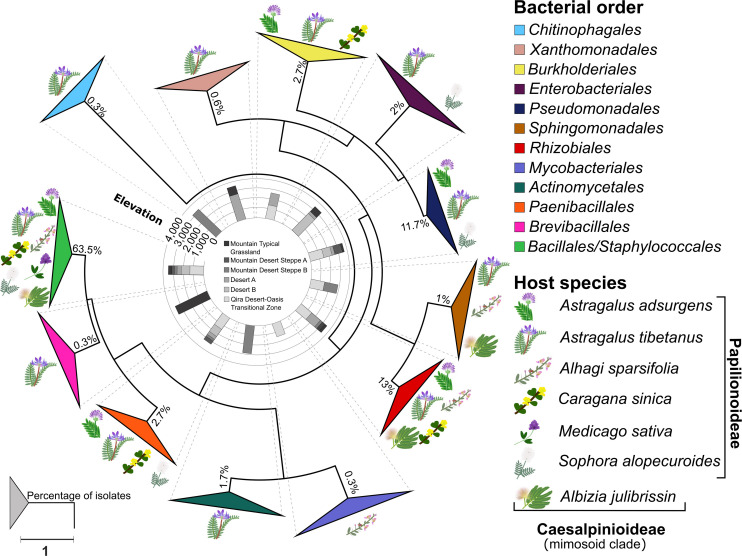
Phylogeny of root nodule-associated bacteria. Maximum-likelihood phylogeny for 16S rRNA gene sequences of all isolates and relevant reference sequences. Groups are collapsed at the order level. Host plant species are indicated on the outside of the collapsed triangles, with graphic representations of the legume hosts. The ecological zone and the associated elevation from which the isolates were obtained are indicated in the middle of the phylogeny. The percentage of isolates belonging to each order is indicated at each node. The scale bar represents the number of nucleotide changes per site. A version of this tree with branches not collapsed is shown in Supplementary Figure 1.

**FIGURE 4:**
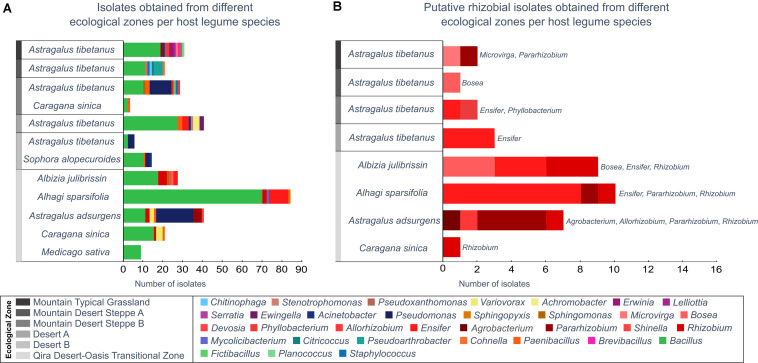
Bacterial isolates belonging to each genus from each ecological zone and host legume species. Genera are ordered in the key according to the phylogeny (Supplementary Figure 1). **(A)** All isolates, as identified based on the 16S rRNA gene maximum-likelihood phylogeny. **(B)** All putative rhizobia (belonging to the *Rhizobiales*), as verified by a *recA* gene phylogeny (Supplementary Figure 2). *Rhizobiales* were only isolated from *Astragalus tibetanus* at higher elevations.

A total of 300 bacterial isolates were isolated from the root nodules and were identified using the 16S rRNA gene. Based on the phylogeny constructed from the 16S rRNA gene sequences, the isolates were assigned to 12 orders (Figure 3 and Supplementary Figure 1): *Actinomycetales*, *Bacillales*, *Burkholderiales*, *Brevibacillales*, *Chitinophagales*, *Enterobacteriales*, *Mycobacteriales*, *Paenibacillales*, *Pseudomonadales*, *Rhizobiales*, *Sphingomonadales*, *Staphylococcales*, and *Xanthomonadales*. Of these, the *Bacillales* (63.5% of all isolates) was the only order that was isolated from all host plants and from each ecological zone (Figure 4A), consisting of a total of 190 isolates, followed by the *Rhizobiales* (13% of all isolates; 39 isolates) and the *Pseudomonadales* (11.7% of all isolates; 35 isolates). The remaining isolates were divided into nine bacterial orders, and the number of isolates in each group was less than 3% of all the isolates (Figure 4A and Supplementary Table 1).

Although the phylogeny did not suggest strong ecological patterning at the order level by either host legume species or ecological zone, a few elevation-specific patterns emerged within the *Bacillales* (Supplementary Table 1 and Supplementary Figure 6) and *Pseudomonadales* (Supplementary Table 1 and Supplementary Figure 7). Within the *Bacillales*, the genus *Bacillus* was dominant, accounting for 186 of the 190 isolates assigned to this order. *Bacillus* isolates were diverse and revealed few ecological patterns. One exception was a strongly supported, monophyletic group of six isolates most closely related to *Bacillus psychrosaccharolyticus* from *A. tibetanus* nodules collected from the highest elevation sites (>3,500 m) within the Mountain Typical Grassland. *A. tibetanus* was sampled from five of the six ecological zones, accounting for 15 separate plants, yet *Bacillus psychrosaccharolyticus* was not isolated from any other ecological zone. In contrast, a strongly supported group of 15 isolates related to *Bacillus endophyticus* and *Bacillus filamentosus* was isolated from all five legume species sampled from the low-elevation Qira Desert-Oasis Transition Zone (1,350 m). Some other groups of *Bacillus* isolates were predominantly isolated from low-elevation sites (e.g., relatives of *Bacillus cereus*), although those groups also included some isolates from higher elevations or were not strongly supported in the phylogeny.

*Pseudomonadales* were isolated from the root nodules of three different host legumes belonging only to the *Papilionoideae*, growing between 1,350 and 3,000 m. *Pseudomonadales* isolates were restricted to the genera *Pseudomonas* (32 isolates) and *Acinetobacter* (three isolates) (Supplementary Table 1 and Supplementary Figure 7), and nearly all formed a monophyletic clade related to *Pseudomonas brassicacearum*, although with low bootstrap support. These strains were isolated mainly from *Astragalus* species, including both *A. adsurgens* in the low-elevation Qira Desert-Oasis Transition Zone and *A. tibetanus* at higher elevations, but two isolates were obtained from *Sophora alopecuroides*. Although the genus *Astragalus* occurs in all ecological zones, *P. brassicacearum* was not isolated from the high-elevation Mountain Desert Steppe A or Mountain Typical Grassland ecological zones (11 total plants), suggesting an elevation limit within the Kunlun Mountains of ∼3,200 m.

Among the 300 isolates, 39 belonged to the *Rhizobiales* and were studied as potential rhizobia (Figure 4B and Supplementary Figures 8, 9). Isolates belonging to the *Rhizobiales* were recovered from nodules obtained from five different legume hosts, sampled between 1,350 and 3,500 m, from five different ecological zones (all except Desert B). The identity of 36 of these isolates was checked through sequencing of the *recA* gene, and 35 were confirmed as members of the *Rhizobiales* (Supplementary Table 1 and Supplementary Figure 9). *Rhizobiales* isolated from five different host plants in the low-elevation Qira Desert-Oasis Transition Zone (1,350–1,960 m) suggested some patterns of host preference. For example, most isolates from *Albizia julibrissin* formed a monophyletic group related to *Rhizobium lemnae* and most from *Alhagi sparsifolia* were closely related to *Ensifer kummerowiae*.

### Key Phenotypic Characteristics of Potential Rhizobia

The 39 *Rhizobiales* isolates were tested for key phenotypic traits. All strains grew well on Ashby’s Mannitol Agar (Hindersah and Perdanawati, 2018), which is a nitrogen-free medium, suggesting they can fix atmospheric nitrogen (Supplementary Tables 1, 4). All strains grew well at 25°C, but were not capable of growth at 60°C (Supplementary Tables 1, 5). Most (8/11) of the strains isolated from ecological zones at middle to high elevation (>2,600 m) were capable of growth at 4°C, whereas only about half (15/28) from the low-elevation Qira Desert-Oasis Transition Zone grew at 4°C. In general, the strains had broad heterotrophic activity (Supplementary Tables 1, 6), with a large number of strains using maltose (35/39), sucrose (34/39), inositol (35/39), and starch (30/39). In contrast, very few isolates used fructose (7/39) and no strains used propionate. Most strains were able to grow with single L-amino acids or ammonium as the sole nitrogen source; however, very few (2/39) used nitrate (Supplementary Tables 1, 7). All tested strains grew well on 3% NaCl, while only a few strains grew well at 7–8% NaCl (Supplementary Table 8). Nearly all strains had a pH growth range of 6–10 (Supplementary Table 9).

## Discussion

Given the extreme conditions of southwestern Xinjiang Province and the availability of a steep elevation gradient instrumented with climate monitoring stations in the Kunlun Mountains, we explored the diversity of legume root nodule-associated bacteria in this system. We sampled seven different legume species from the legume subfamilies *Papilionoideae* and *Caesalpinioideae*, from six broadly defined ecological zones. Our results revealed a high diversity among the 300 isolates, belonging to 32 genera and 12 orders. In comparison, De Meyer et al., 2015 reported 51 genera among 654 endophyte isolates from 30 plant species in Belgium and Xu et al., 2014 reported 35 genera among 201 endophyte isolates from two plant species in China.

It has previously been documented that many non-rhizobial endophytes are often associated with root nodules of a variety of legumes (Dudeja et al., 2012; Xu et al., 2014; De Meyer et al., 2015) and the genetic diversity of these endophytes is often high (Dudeja et al., 2012; De Meyer et al., 2015). Among these, *Bacillus* and *Pseudomonas* are particularly common (Dudeja et al., 2012; De Meyer et al., 2015) and these genera are well-recognized for their roles in plant growth promotion and biocontrol over soil-borne pathogens (Santoyo et al., 2012). These two genera are also prominent among rhizoplane bacteria of a variety of plants. Thus, the high diversity of root nodule-associated bacteria in this study and the predominance of *Bacillus* and *Pseudomonas* was not unexpected.

Although a variety of climatic and edaphic factors varied over this elevation gradient, soil temperature, and conductivity are known to be important primary controls on microbial diversity, community structure, and community function (Lozupone and Knight, 2007; Sunagawa et al., 2015) and were likely to be important in structuring the bacterial communities studied here. The current study suggested limited relationships between the phylogenetic distribution of root nodule-associated bacteria and the ecological zone from which they were isolated, with the most promising patterns being the apparent restriction of *B. psychrosaccharolyticus* relatives to the highest elevations (>3,500 m), the apparent restriction of *B. endophyticus* relatives to the lowest elevations (>1,300 m), and the apparent restriction of *P. brassicacearum* relatives to middle and low elevations (<3,200 m). *B. psychrosaccharolyticus* is psychrotrophic and can sporulate and grow at 0°C (Larkin and Stokes, 1967), although it is not commonly known as an endophyte (Logan and de Vos, 2015). We speculate that the low soil temperatures at the high elevations in the Kunlun Mountains may select against mesophilic *Bacillus* strains, leaving an open niche for *B. psychrosaccharolyticus* within the root nodules. Of note, many *Bacillus* species cannot grow below 10°C (Logan and de Vos, 2015), and the highest monthly soil temperature recorded in the Mountain Typical Grassland soils was only 12.01°C (Figure 2 and Supplementary Table 3). Most *Bacillus* isolates from *A. tibetanus* were closely related to *Bacillus licheniformis*, which are well-known endophytes, but have a minimum growth temperature of 15°C. *Bacillus endophyticus* relatives were isolated from all five legume species within the low-elevation Qira Desert-Oasis Transition Zone but no other ecological zones. *B. endophyticus* is a common endophyte of a variety of plants, but the type strain does not grow below 10°C (Reva et al., 2002). Thus, soil temperatures may be a dominant ecological driver for *Bacillus* strains associated with root nodules in this study.

*Pseudomonas brassicacearum* relatives were the dominant *Pseudomonadales* isolates predominantly from *Astragalus* species and appeared to be restricted to middle and low elevations (<3,200 m), as was the genus *Pseudomonas* in general. *P. brassicacearum* is a well-known plant growth-promoting endophyte within the *Pseudomonas fluorescens* group (Gislason and de Kievit, 2020), but the characterized strains of *P. brassicacearum* do grow at 4°C, so soil temperature may not be a direct driver of its distribution. A study of wild legumes in high-elevation grasslands on the Tibetan Plateau (∼2,900 m) also failed to recover *Pseudomonas* endophytes, and the authors similarly suggested that this ecosystem does not favor *Pseudomonas* endophytes (Wang et al., 2016). In contrast, in a separate study of alpine mosses in the Qilian Mountains in the Qinghai-Tibet Plateau, *Pseudomonas* strains accounted for over half of the isolates (Lan et al., 2020).

Few studies have directly addressed relationships between elevation and bacterial endophytes of legume nodules. However, elevation is known to correlate with the composition and/or diversity of fungal endophytes of native plants. For example, a cultivation-independent study showed that middle elevations hosted the lowest diversity of fungal endophytes along an elevational transect on Mauna Loa Volcano, Hawaii (Cobian et al., 2019). Although elevation was not observed to correlate with community structure in that study, an earlier study along the same elevation gradient did report elevation as a strong correlate of microbial community structure (Zimmerman and Vitousek, 2012). Given the strong elevational trends for both climatic and edaphic data, and well-known elevational trends in flora and fauna in the Kunlun Mountains, some relationships between elevation and root nodule-associated bacteria are not surprising.

In a separate cultivation-independent study of the biogeography of nodule endophytes in soybeans across China, the rhizobia subcommunities, mainly composed of *Bradyrhizobium* and *Ensifer*, were significantly correlated with soil pH, with *Bradyrhizobium* dominating in alkaline soils in central China and *Ensifer* dominating in neutral and acidic soils in northeast and southern China (Zhang et al., 2018). Other studies have also shown that soil pH is more important than nutrient content in shaping microbial communities in agricultural settings (Wang et al., 2019), in addition to soil inorganic nitrogen (Zhao et al., 2018). In the current study, however, biases associated with plant sampling and microbial isolation preclude a strong framework to quantitatively test relationships between bacterial populations and climatic and edaphic factors. The large diversity of ecological zones, host plants, and microbial isolates that were studied also complicates potential relationships between these factors and particular bacterial taxa. Finally, the limited distribution of legume species along the elevation gradient limited insight into the effects of ecological zones on nodule-associated bacteria. Ultimately, a cultivation-independent approach, though biased, would provide a stronger framework for testing possible factors controlling the diversity and composition of nodule-associated bacterial communities.

Among the isolates, several putative rhizobial genera were identified: *Agrobacterium*, *Allorhizobium*, *Bosea*, *Ensifer*, *Microvirga*, *Pararhizobium*, *Phyllobacterium*, and *Rhizobium*. The putative rhizobia consumed a variety of organic substrates and showed evidence of nitrogen fixation, with some tolerance to NaCl and pH extremes. In recent research, diverse rhizobia including *Bradyrhizobium*, *Ensifer*, and *Rhizobium* were identified from soybean nodules collected from Xinjiang Province, where soybean is an exotic plant (Han et al., 2009). A separate study assessed the genetic diversity of *Caragana* nodule rhizobial isolates collected from arid and semi-arid alkaline sandy soils in the north of China, revealing *Bradyrhizobium*, *Mesorhizobium*, and *Rhizobium* as the predominant genera (Li et al., 2012). Based on our studies, *Rhizobium* and *Ensifer* appear to be widespread and potentially abundant rhizobia populations in the Kunlun Mountains. The absence of *Bradyrhizobium* from this collection of isolates was conspicuous. Although *Bradyrhizobium* should be able to grow under the conditions used here, they are typically slow-growing, and it is possible that they were missed by our cultivation approach due to slow growth or were lost during the isolation procedure. Thus, the current study certainly does not rule out an important role for *Bradyrhizobium* and other fastidious or slow-growing endophytes in this system.

In the current study, a variety of potential rhizobia were isolated from nodules from legumes in the low-elevation Qira Desert-Oasis Transition Zone, and a few patterns suggested host preference: *Rhizobium lemnae* relatives from *Albizia julibrissin* and *Ensifer kummerowiae* relatives from *Alhagi sparsifolia*. A previous study of root nodule endophytes within several *Albizia* species and colocalized *Acacia* and *Leuceana* species in China uncovered patterns of host preference, with *Bradyrhizobium*, *Mesorhizobium*, and *Rhizobium* strains being isolated from *Albizia* (Wang et al., 2016). Thus, the isolation of a distinct clade of *Rhizobium* from *Albizia* in this study is not unexpected, although the isolation of a few strains of *Bosea*, *Devosia*, and *Ensifer* from *Albizia* may be unique. *Alhagi* species are often nodulated by *Mesorhizobium* or *Rhizobium* (Andrews and Andrews, 2017), so the predominance of *Ensifer* strains, described here, may be unusual. Similarly, *Mesorhizobium* is commonly isolated from *Astragalus adsurgens* (Gao et al., 2004), although *Pararhizobium* isolates have previously been described from other species of *Astragalus.* Since most of the legume diversity was only present in that ecological zone, our ability to explore potential relationships between root nodule inhabitants within the elevational/ecological gradient was limited. In general, the interaction among host species, rhizobial host range, and environmental variation determine the structure of rhizobial communities, although these relationships are complex and not clearly predictable (Vuong et al., 2017).

In contrast to the limited distribution of most of the legumes along the elevation gradient, *Astragalus tibetanus* occurred in five ecological zones, providing an opportunity to examine relationships between elevation and rhizobia present within the nodules of a single legume species. However, the plants sampled in those five zones hosted a wide variety of *Rhizobiales* genera, including *Ensifer, Bosea*, *Microvirga*, *Phyllobacterium*, and *Pararhizobium* and a relatively small number of isolates prevented an assessment of relationships between elevation and rhizobia in this plant.

Overall, the high diversity in nodule-associated bacteria, including putative rhizobia, may provide the legumes in the Kunlun Mountains with better capabilities to adapt to extreme and variable environmental conditions. Although this study was complex, it suggests that edaphic and/or climatic variables may control the distribution of nodule bacteria within the context of the extremes studied here.

## Data Availability Statement

The original contributions presented in the study are publicly available. These data can be found in GenBank under accession numbers MT634394-MT634690 (Supplementary Table 1), MW306700-MW306735, and MW396711: https://www.ncbi. nlm.nih.gov/nuccore/MT634394.1/, https://www.ncbi.nlm.nih. gov/nuccore/MW396711, and https://www.ncbi.nlm.nih.gov/search/all/?term=MW306700.

## Author Contributions

JP and HS participated in the design of the study. JP performed all the experiments and wrote the initial drafts of the manuscript. MP helped with phylogenetic analyses, interpretation of results, revising initial drafts, and polishing figures. LZ contributed to the isolation and identification of endophytic bacteria and the characterization of potential plant-beneficial traits. MP and CS did the statistical analyses and data analysis. BH, MP, and FZ helped to revise the manuscript and supervised laboratory experiments. All authors edited and critically revised the manuscript.

## Conflict of Interest

The authors declare that the research was conducted in the absence of any commercial or financial relationships that could be construed as a potential conflict of interest.

## Footnotes

1 http://cld.cern.ac.cn/meta/metaData

2 http://www.eztaxon.org

## Supplementary Material

Supplementary Figure 1 Rhizosphere soil characteristics measured across the different ecological zones. The bars represent the mean ± S.D. for all plants sampled in each ecological zone. There were no significant relationships (Spearman’s rho *p* < 0.05) between elevation and any of the soil chemistry measurements. Distinct letters are significantly different (ANOVA *p* < 0.05).

Supplementary Figure 2 Mean monthly atmospheric temperatures measured at climate monitoring stations in the different ecological zones. The bars represent the mean ± S.D. for all data collected for a given month. Asterisks next to months indicate a significant relationship (Spearman’s rho *p* < 0.05) between elevation and atmospheric temperature. Distinct letters are significantly different (ANOVA *p* < 0.05).

Supplementary Figure 3 Mean monthly soil volumetric water content measured at climate monitoring stations in the different ecological zones. The bars represent the mean ± S.D. for all data collected for a given month. Asterisks next to months indicate a significant relationship (Spearman’s rho *p* < 0.05) between elevation and soil volumetric water content. Distinct letters are significantly different (ANOVA *p* < 0.05).

Supplementary Figure 4 Mean monthly soil relative humidity measured at climate monitoring stations in the different ecological zones. The bars represent the mean ± S.D. for all data collected for a given month. Asterisks next to months indicate a significant relationship (Spearman’s rho *p* < 0.05) between elevation and soil relative humidity. Distinct letters are significantly different (ANOVA *p* < 0.05).

Supplementary Figure 5 Circular phylogeny of all isolated root nodule strains. Multiple sequence alignments of the 16S rRNA gene sequence for all 300 isolates and appropriate reference sequences were constructed with the SINA aligner v. 1.2.11 and subjected to maximum-likelihood analysis with RAxML v. 8.2.12. Genera are colored based on the orders to which they belong. Branch support was inferred from 1,000 replicates, with branches with support values >70% indicated in black and branches with support values <70% indicated in gray. The scale bar indicates the number of nucleotide changes per site.

Supplementary Figure 6 Circular phylogeny of *Bacillales* strains reprojected from the global phylogeny (Supplementary Figure 5). Next to each strain number is the Latin name for the host plant, colored according to the species, the unique plant identifier in brackets (see Supplementary Tables 1, 2), and the ecological zone from which the strain was isolated. Other details of the phylogenetic analysis are described in Supplementary Figure 5 and “Materials and Methods.”

Supplementary Figure 7 Phylogeny of *Pseudomonadales* strains reprojected from the global phylogeny (Supplementary Figure 5). Next to each strain number is the Latin name for the host plant, colored according to the species, the unique plant identifier in brackets (see Supplementary Tables 1, 2), and the ecological zone from which the strain was isolated. Other details of the phylogenetic analysis are described in Supplementary Figure 5 and “Materials and Methods.”

Supplementary Figure 8 Circular phylogeny of *Rhizobiales* strains reprojected from the global phylogeny (Supplementary Figure 5). Next to each strain number is the Latin name for the host plant, colored according to the species, the unique plant identifier in brackets (see Supplementary Tables 1, 2), and the ecological zone from which the strain was isolated. Other details of the phylogenetic analysis are described in Supplementary Figure 5 and “Materials and Methods.”

Supplementary Figure 9 Circular phylogeny of all potential rhizobia isolated from root nodules based on the *recA* gene. Multiple sequence alignment was done with MAFFT v. 6.24 and Maximum Likelihood analysis was conducted with RAxML v. 8.2.12. The scale bar indicates the number of nucleotide changes per site. The tree was rooted using *Sphingomonas* as outgroup and branch support was inferred from 1,000 bootstrap replicates, with branches with support values >70% indicated in black and branches with support values <70% indicated in gray.

Supplementary Table 1 Isolate information including sample site, host, genus identification, and phenotypic information.

Supplementary Table 2 Plant information including sample site, host, and rhizosphere soil data used to generate Figure 2.

Supplementary Table 3 Monthly mean climate and edaphic data collected near the sampling locations.

Supplementary Table 4 Summary of growth of *Rhizobiales* isolates on Ashby’s Mannitol Agar medium per ecological zone.

Supplementary Table 5 Summary of growth of *Rhizobiales* isolates at different temperatures per ecological zone.

Supplementary Table 6 Summary of growth of *Rhizobiales* isolates on various carbon sources per ecological zone.

Supplementary Table 7 Summary of growth of *Rhizobiales* isolates on various sole nitrogen sources per ecological zone.

Supplementary Table 8 Summary of salt tolerance permitting growth of *Rhizobiales* isolates per ecological zone.

Supplementary Table 9 Summary of growth at different pH ranges for *Rhizobiales* isolates per ecological zone.
