# Supplementary material for: Diversity of Root Nodule-Associated Bacteria of Diverse Legumes Along an Elevation Gradient in the Kunlun Mountains, China
Source: Front Microbiol. 2021 Feb 16;12:633141. doi: 10.3389/fmicb.2021.633141 (PMC7920992; doi:10.3389/fmicb.2021.633141)
Supplement: Supplementary Figure 1 — Rhizosphere soil characteristics measured across the different ecological zones. The bars represent the mean ± S.D. for all plants sampled in each ecological zone. There were no significant relationships (Spearman’s rho p < 0.05) between elevation and any of the soil chemistry measurements. Distinct letters are significantly different (ANOVA p < 0.05). [file Data_Sheet_1.PDF]

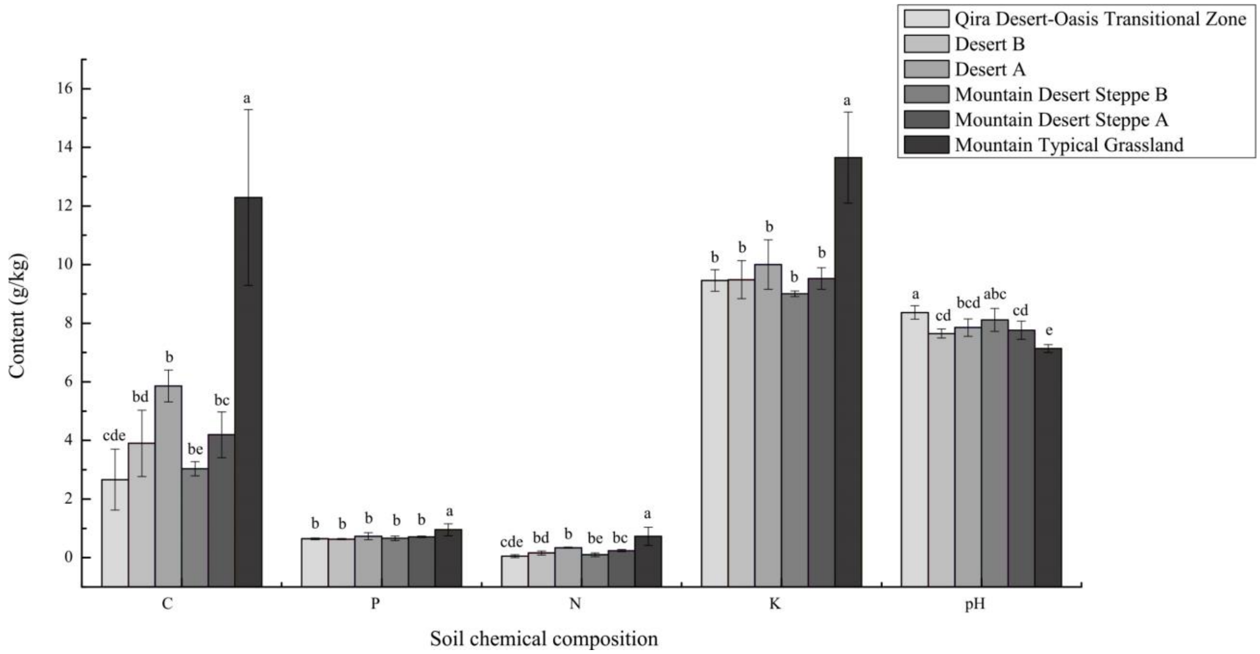

Figure S1. Rhizosphere soil characteristics measured across the different ecological zones. The bars represent the mean ± S.D. for all plants sampled in each ecological zone. There were no significant relationships (Spearman's rho $p<0.05$) between elevation and any of the soil chemistry measurements. Distinct letters are significantly different (ANOVA $p<0.05$).

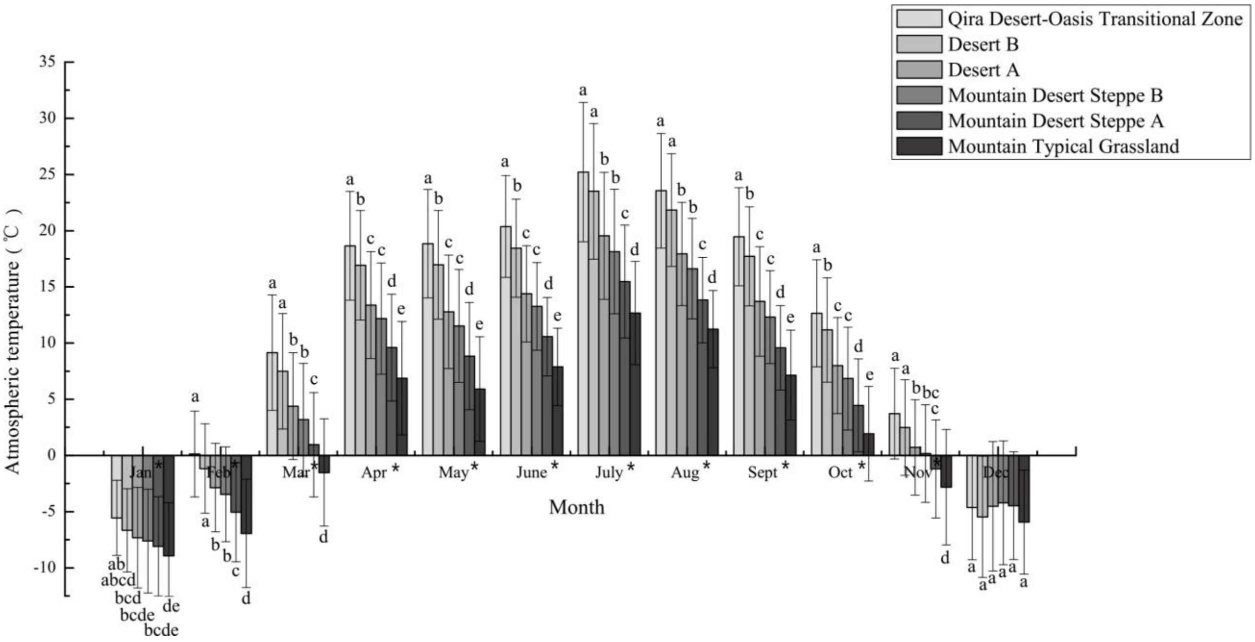

Figure S2. Mean monthly atmospheric temperatures measured at climate monitoring stations in the different ecological zones. The bars represent the mean ± S.D. for all data collected for a given month. Asterisks next to months indicate a significant relationship (Spearman's rho $p<0.05$) between elevation and atmospheric temperature. Distinct letters are significantly different (ANOVA $p<0.05$).

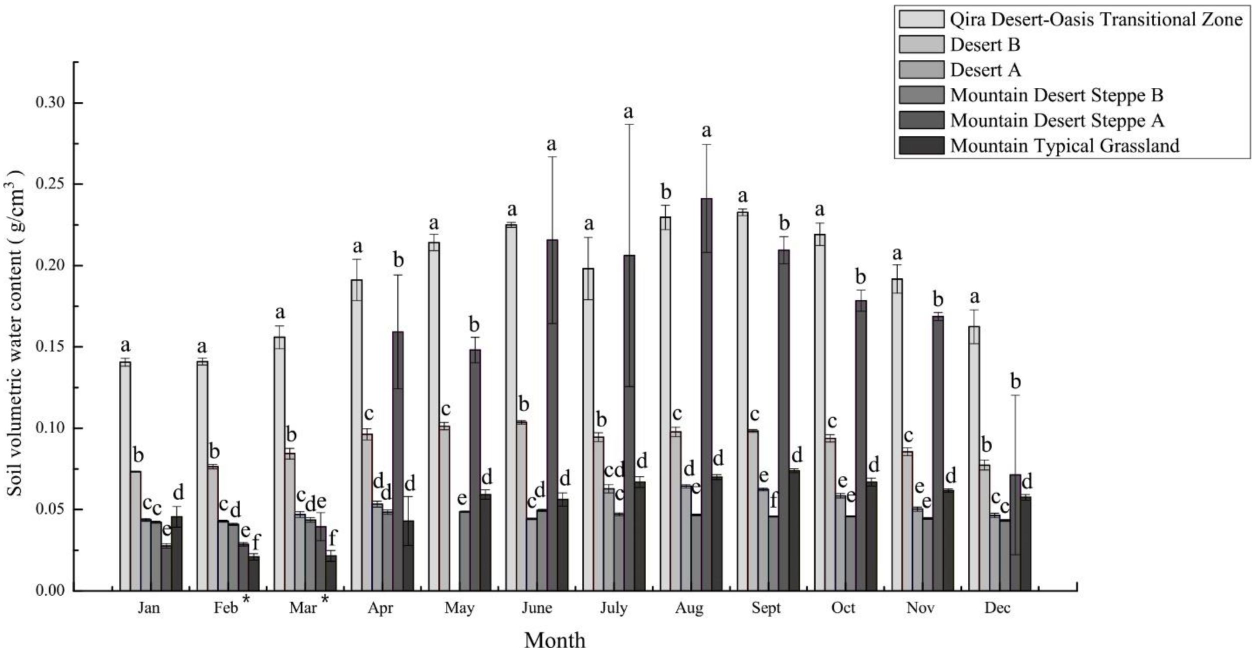

Figure S3. Mean monthly soil volumetric water content measured at climate monitoring stations in the different ecological zones. The bars represent the mean ± S.D. for all data collected for a given month. Asterisks next to months indicate a significant relationship (Spearman's rho $p<0.05$) between elevation and soil volumetric water content. Distinct letters are significantly different (ANOVA $p<0.05$).

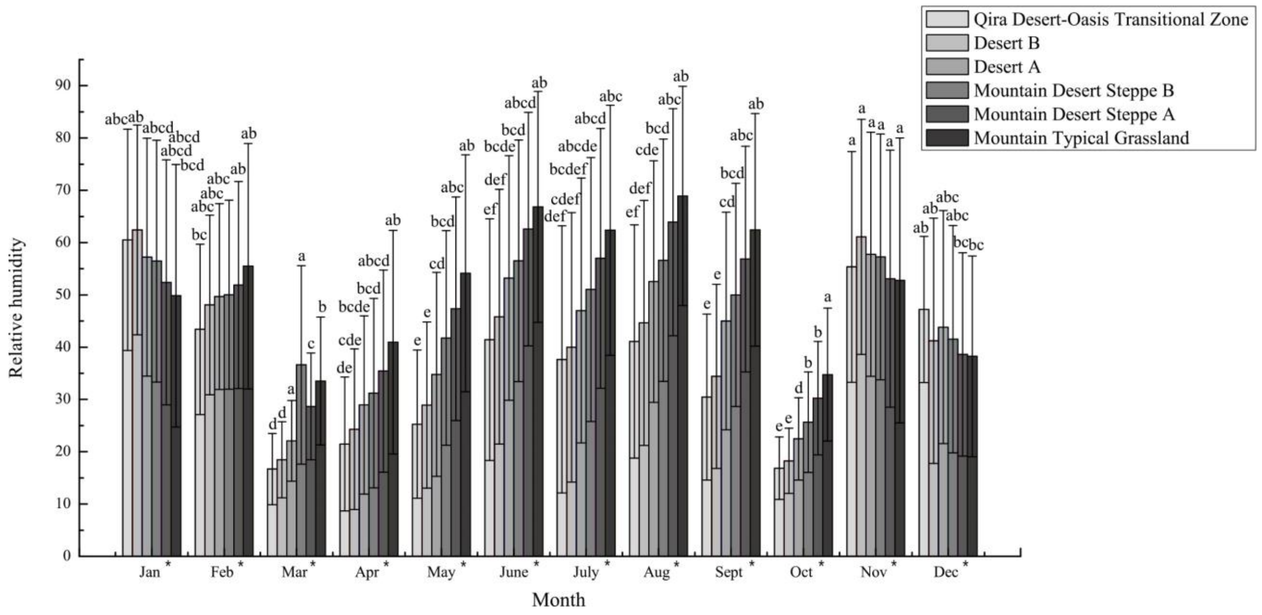

Figure S4. Mean monthly soil relative humidity measured at climate monitoring stations in the different ecological zones. The bars represent the mean ± S.D. for all data collected for a given month. Asterisks next to months indicate a significant relationship (Spearman's rho $p<0.05$) between elevation and soil relative humidity. Distinct letters are significantly different (ANOVA $p<0.05$).

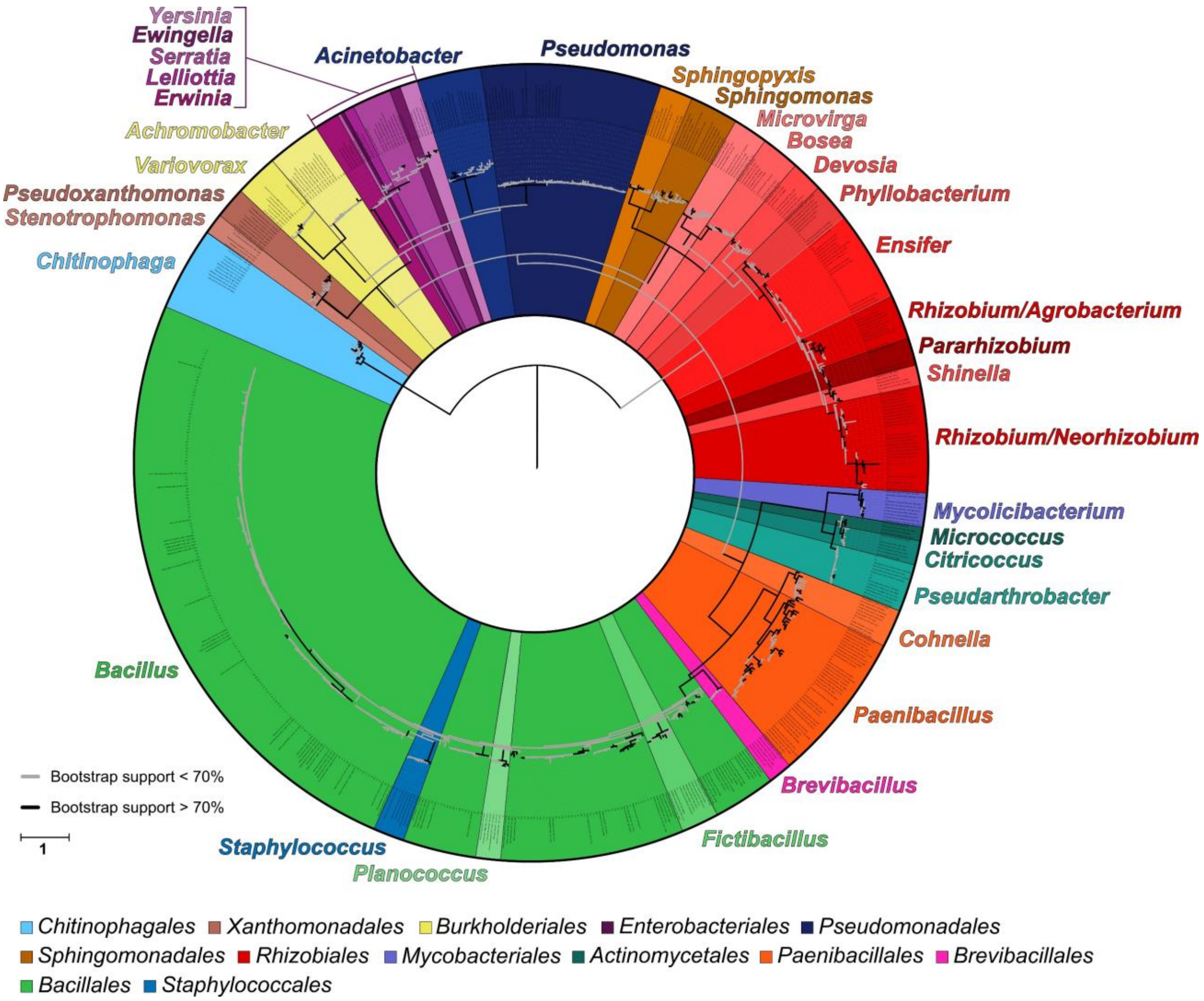

Figure S5. Circular phylogeny of all isolated root nodule strains. Multiple sequence alignments of the 16S rRNA gene sequence for all 300 isolates and appropriate reference sequences were constructed with the SINA aligner v. 1.2.11 and subjected to maximum-likelihood analysis with RAxML v. 8.2.12. Genera are colored based on the orders to which they belong. Branch support was inferred from 1000 replicates, with branches with support values > 70% indicated in black and branches with support values < 70% indicated in grey. The scale bar indicates the number of nucleotide changes per site.

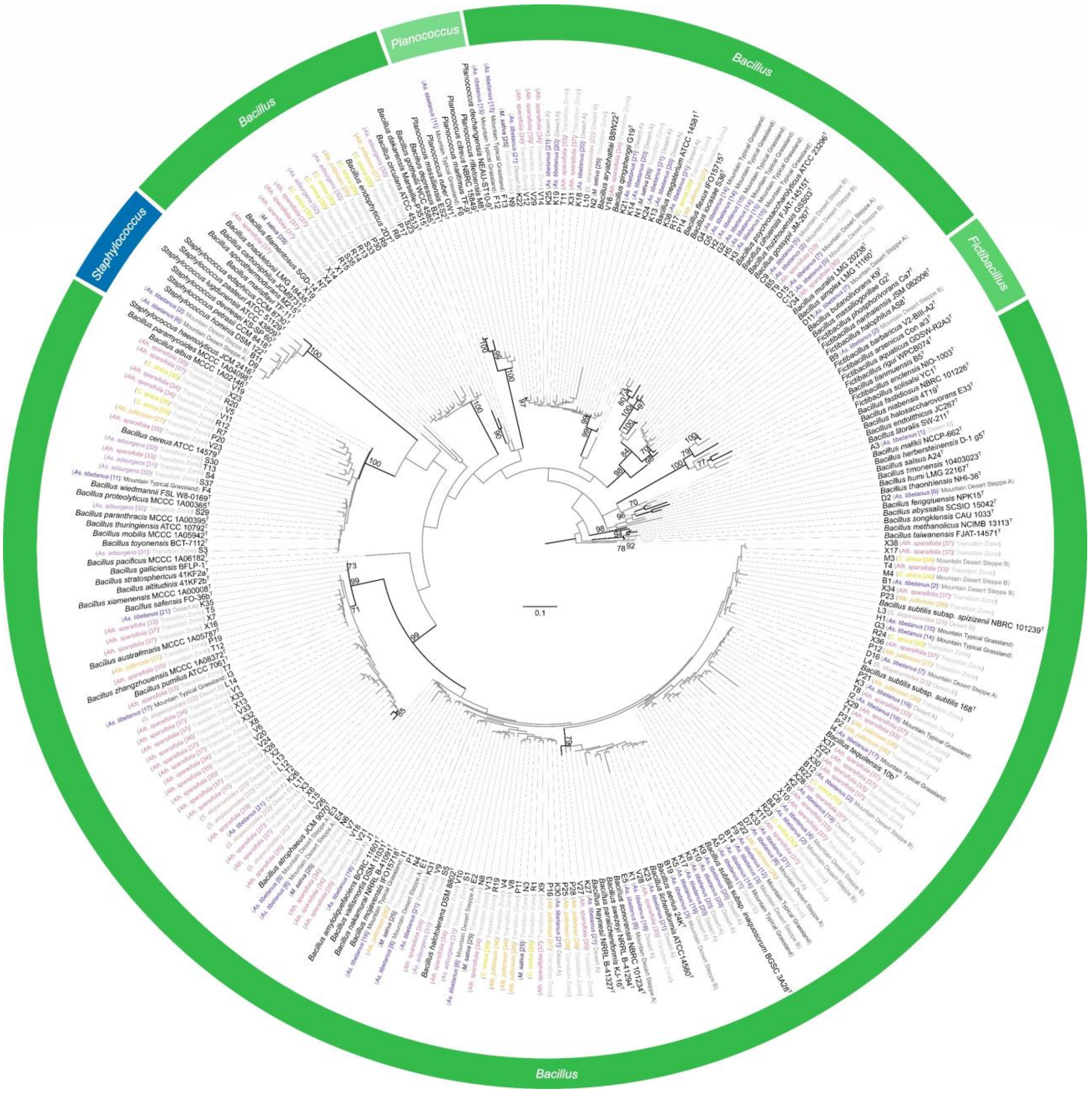

Figure S6. Circular phylogeny of *Bacillales* strains reprojected from the global phylogeny (Figure S5). Next to each strain number is the Latin name for the host plant, colored according to the species, the unique plant identifier in brackets (see Table S1 and Table S2), and the ecological zone from which the strain was isolated. Other details of the phylogenetic analysis are described in Figure S5 and Methods.

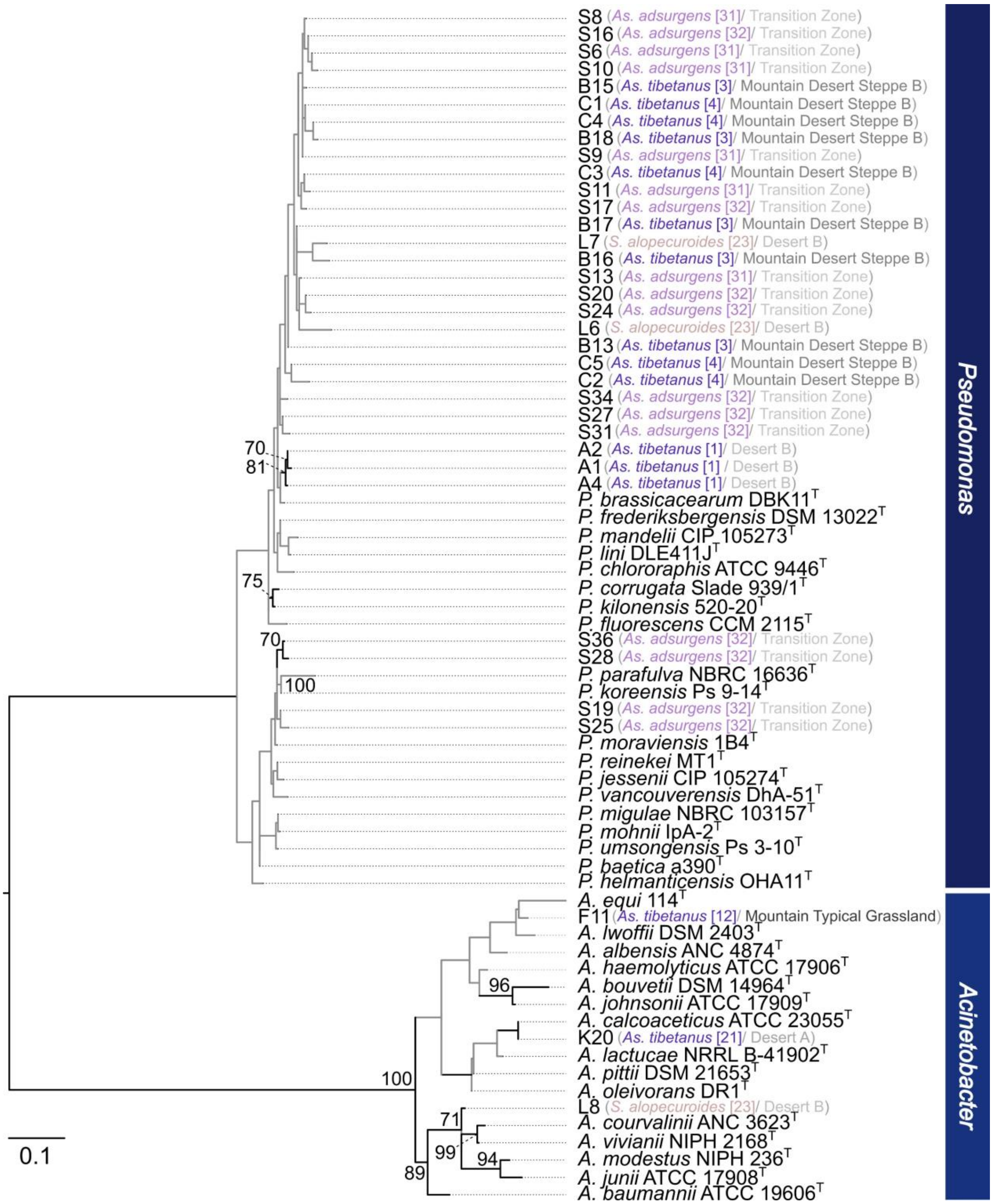

Figure S7. Circular phylogeny of *Pseudomonadales* strains reprojected from the global phylogeny (Figure S5). Next to each strain number is the Latin name for the host plant, colored according to the species, the unique plant identifier in brackets (see Table S1 and Table S2), and the ecological zone from which the strain was isolated. Other details of the phylogenetic analysis are described in Figure S5 and Methods.

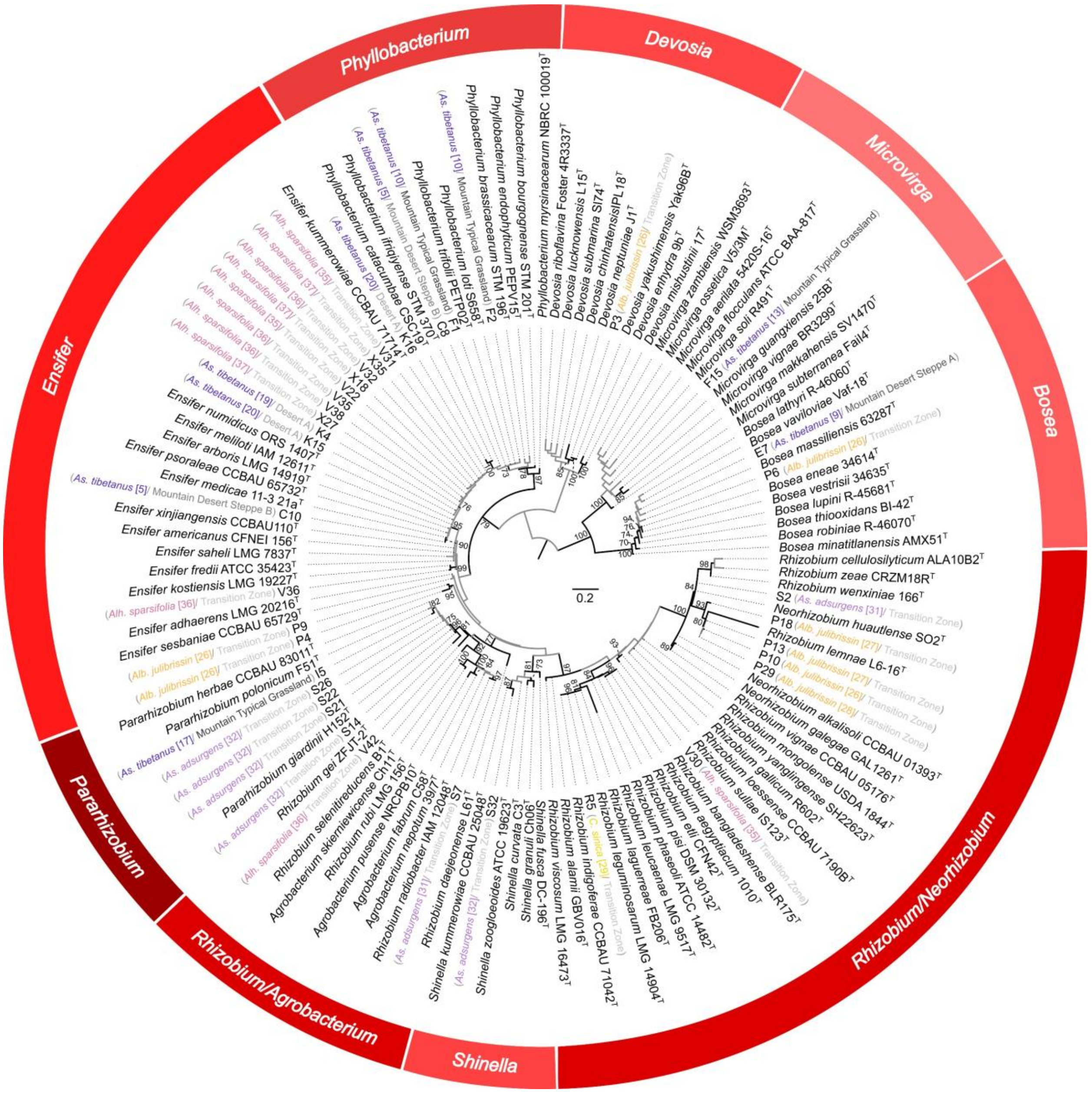

Figure S8. Circular phylogeny of *Rhizobiales* strains reprojected from the global phylogeny (Figure S5). Next to each strain number is the Latin name for the host plant, colored according to the species, the unique plant identifier in brackets (see Table S1 and Table S2), and the ecological zone from which the strain was isolated. Other details of the phylogenetic analysis are described in Figure S5 and Methods.

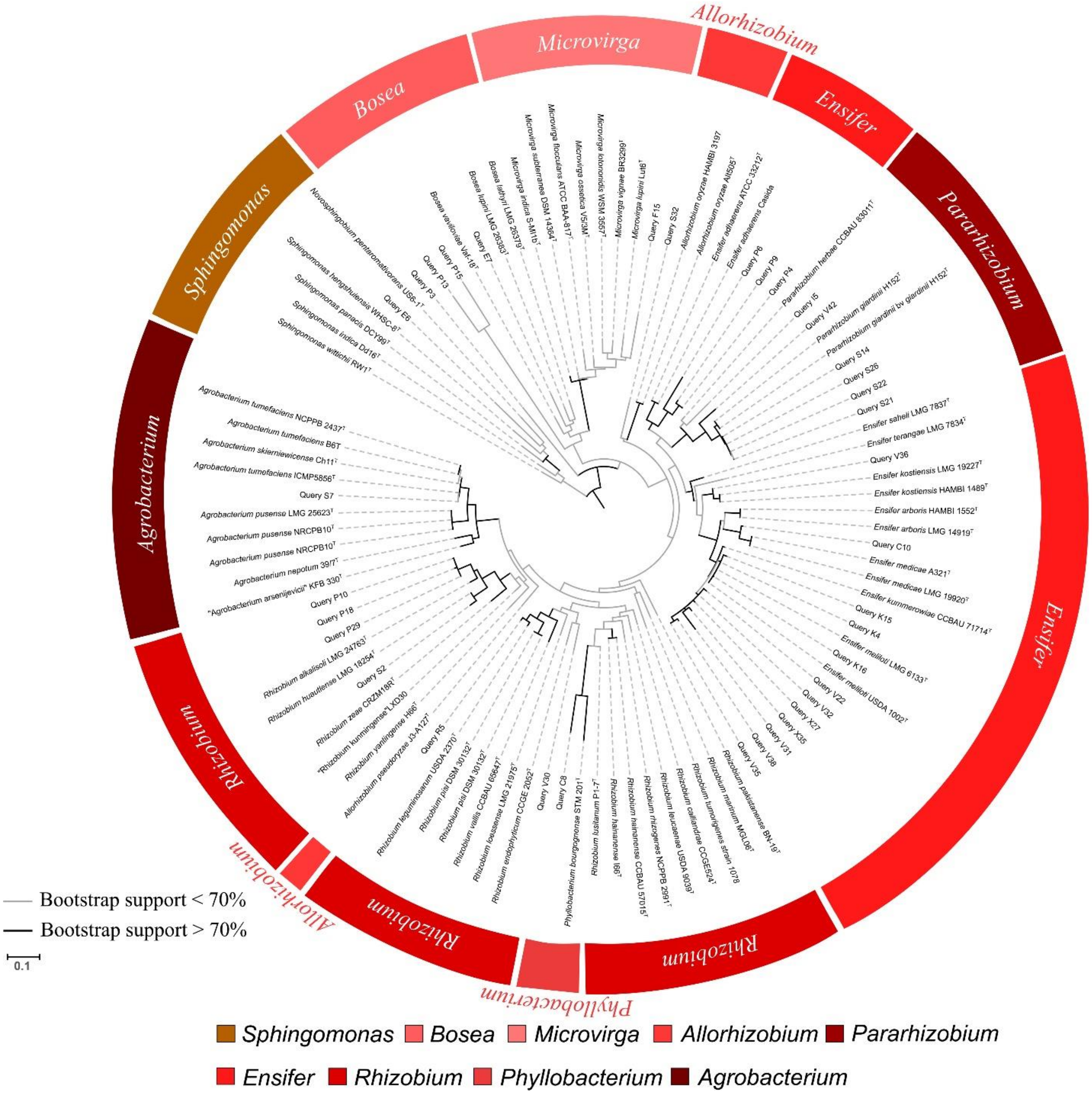

Figure S9. Circular phylogeny of all potential rhizobia isolated from root nodules based on the *recA* gene. Multiple sequence alignment was done with MAFFT v. 6.24 and maximum likelihood analysis was conducted with RAxML v. 8.2.12. The scale bar indicates the number of nucleotide changes per site. The tree was rooted using *Sphingomonas* as outgroup and branch support was inferred from 1000 bootstrap replicates, with branches with support values > 70% indicated in black and branches with support values < 70% indicated in grey.
